# 

**Published:** 1919-04

**Authors:** 


### CONTENTS FOR THE APRIL ISSUE

EDITORIALS

Looking to the Future.......... 329

Sleeping Sickness.......... 330

Medical Colleges.......... 331

Health Resorts and Outing Places.......... 331

The Medical Letter Bag.......... 331

Dr. W. D. Yett, Mayor.......... 332

Hospitals.......... 333

ORIGINAL ARTICLES

The Treatment of Congenital Club-foot.......... 334

The Biological and Therapeutic and the Clinical Value of Radium and Roentgen Rays.......... 340

Preservation of the Uterus in Removal of Chronic Infected Adnexa.......... 348

The Medical Letter Bag.......... 354

Sleeping Sickness.......... 357

Texas State Medical Convention.......... 361

New Advertisers.......... 364

Army News.......... 366

Old Jokes We Have Met.......... 370

